# Targeting the Sleep–Glymphatic–Vascular Continuum in Cerebral Small Vessel Disease: A Nutritional Perspective on Neuroprotective Potential of Tocotrienols (T3)

**DOI:** 10.3390/life16030393

**Published:** 2026-02-28

**Authors:** Dena Farysah Mazli, Zaw Myo Hein, Che Mohd Nasril Che Mohd Nassir, Ain Hafizah Alias, Sint Sint Win, Mohammad Farris Iman Leong Abdullah, Muhammad Zulfadli Mehat, Hafizah Abdul Hamid, Gehan El-Akabawy

**Affiliations:** 1 Department of Human Anatomy, Faculty of Medicine and Health Sciences, Universiti Putra Malaysia, Serdang 43400, Selangor, Malaysia; gs73842@student.upm.edu.my (D.F.M.); gs69187@student.upm.edu.my (A.H.A.); m_zulfadli@upm.edu.my (M.Z.M.); 2 Department of Basic Medical Sciences, College of Medicine, Ajman University, Ajman P.O. Box 346, United Arab Emirates; z.hein@ajman.ac.ae; 3 Centre of Medical and Bio-Allied Health Sciences Research, Ajman University, Ajman P.O. Box 346, United Arab Emirates; 4 Department of Anatomy and Physiology, Faculty of Medicine, Universiti Sultan Zainal Abidin, Kuala Terengganu 20400, Terengganu, Malaysia; nasrilnassir@unisza.edu.my; 5 Department of Internal Medicine, University of Medicine 1, Yangon, No. 245, Myoma-Kyaung Street, Lanmadaw Township, Yangon 11131, Myanmar; sintsint994@gmail.com; 6 Department of Psychiatry, Faculty of Medicine, Universiti Sultan Zainal Abidin, Kuala Terengganu 20400, Terengganu, Malaysia; farrisiman@unisza.edu.my; 7 Department of Anatomy and Embryology, Faculty of Medicine, Menoufia University, Menofia 6132720, Egypt

**Keywords:** cerebral small vessel disease, glymphatic system, sleep fragmentation, vitamin E, tocotrienols, neuroprotection

## Abstract

Cerebral small vessel disease (CSVD) is a leading cause of stroke, cognitive impairment, and vascular dementia, yet disease-modifying therapeutic strategies remain limited. Emerging evidence suggests that sleep fragmentation (SF), a common and often under-recognized feature of aging and cardiometabolic disorders, plays a pivotal role in CSVD pathogenesis by disrupting the glymphatic system, the brain’s primary waste clearance pathway. Sleep-dependent glymphatic function facilitates the removal of neurotoxic metabolites and maintains neurovascular homeostasis. In contrast, SF impairs cerebrospinal fluid (CSF)–interstitial fluid (ISF) exchange, promotes perivascular space enlargement, endothelial dysfunction, blood–brain barrier (BBB) breakdown, and chronic neuroinflammation, hallmarks of CSVD. This review synthesizes current mechanistic, preclinical, and clinical evidence linking SF to glymphatic dysfunction and small vessel pathology, framing these interactions as a sleep–glymphatic–vascular continuum underlying CSVD progression and cognitive decline. We further explore the emerging therapeutic potential of tocotrienols (T3), vitamin E isoforms with potent antioxidant, anti-inflammatory, and vasculoprotective properties, as modulators of neurovascular integrity within this continuum. Although direct evidence linking T3 to glymphatic regulation remains limited, converging data support their capacity to preserve endothelial function, attenuate oxidative stress, and stabilize astrocytic and BBB dynamics, mechanisms highly relevant to glymphatic and microvascular health. By integrating sleep biology, glymphatic neuroscience, and nutritional vascular protection, this review highlights hypothesis-generating preventive and therapeutic avenues for CSVD and delineates key knowledge gaps, including the need for longitudinal human studies, standardized glymphatic imaging, objective sleep phenotyping, and interventional trials to establish causal and translational relevance.

## 1. Introduction

Cerebral small vessel disease (CSVD) is a highly prevalent, age-associated cerebrovascular disorder and a leading contributor to stroke, cognitive impairment, gait disturbance, and vascular dementia worldwide [1,2]. Neuroimaging markers of CSVD, including white matter hyperintensities (WMHs), lacunes, cerebral microbleeds (CMBs), and enlarged perivascular spaces (ePVS), are detected in more than 80% of individuals over the age of 70, underscoring its substantial public health burden [3,4]. Beyond aging, CSVD is strongly influenced by modifiable lifestyle and metabolic factors, including hypertension, diabetes, obesity, and increasingly recognized behavioral determinants such as sleep quality [5,6,7]. Despite its high prevalence and cumulative impact on cognitive and functional decline [8,9], effective disease-modifying strategies for CSVD remain limited, highlighting the importance of preventive approaches targeting early and modifiable biological pathways.

Sleep fragmentation (SF), characterized by frequent arousals and disruption of normal sleep architecture, is highly prevalent in modern societies and disproportionately affects older adults and individuals with cardiometabolic disorders [10]. Epidemiological studies have linked SF to increased risks of stroke, vascular cognitive impairment, and accelerated brain aging, independent of traditional vascular risk factors [11,12]. From a nutritional and metabolic perspective, sleep disruption is closely intertwined with oxidative stress, systemic inflammation, endothelial dysfunction, and impaired glucose and lipid metabolism [13,14], all processes central to CSVD pathophysiology. These observations suggest that SF may act as a modifiable upstream driver of neurovascular injury, offering an opportunity for lifestyle and nutrient-based interventions.

Additionally, the discovery of the glymphatic system has provided a mechanistic bridge linking sleep quality to brain metabolic homeostasis [7,15]. This astrocyte-dependent perivascular clearance pathway facilitates the exchange of cerebrospinal fluid (CSF) and interstitial fluid (ISF), enabling the removal of metabolic waste products, neurotoxic proteins, and inflammatory mediators [15,16]. Despite the glymphatic system being a widely discussed but still evolving framework for brain-wide waste clearance, with ongoing debate regarding its dominant driving forces, methodological artifacts in its measurement, and the extent to which rodent findings translate to human brain physiology. Moreover, glymphatic activity is strongly enhanced during non–rapid eye movement (NREM) slow-wave sleep (SWS) and is markedly impaired by SF [17], and disrupted glymphatic function have been suggested to lead to ePVS, blood–brain barrier (BBB) dysfunction, and chronic neuroinflammation, key pathological features increasingly implicated in CSVD and age-related cognitive decline [18].

Within this framework, nutrition has emerged as a critical modulator of neurovascular and glymphatic health. Among bioactive nutrients, tocotrienols (T3), members of the vitamin E family predominantly found in palm oil, annatto, and rice bran, exhibit potent antioxidant, anti-inflammatory, and vasculoprotective properties that extend beyond those of tocopherols [19]. Preclinical and clinical evidence suggest that T3 may improve endothelial function, attenuate oxidative stress, preserve BBB integrity, and modulate glial activation, mechanisms highly relevant to both glymphatic efficiency and small vessel integrity [20]. These properties position T3 as a promising nutritional candidate for mitigating sleep-related neurovascular dysfunction.

This review critically synthesizes current epidemiological, mechanistic, and translational evidence linking SF, glymphatic dysfunction, and CSVD, conceptualizing these interactions as a unified sleep–glymphatic–vascular continuum. This review emphasizes the emerging role of T3 as a nutraceutical strategy to support neurovascular resilience, identify key knowledge gaps, and discuss future directions for nutrition-based interventions targeting sleep-dependent brain health and CSVD prevention.

## 2. Sleep Architecture and Fragmentation

### 2.1. Normal Sleep Physiology

**Figure 1:**
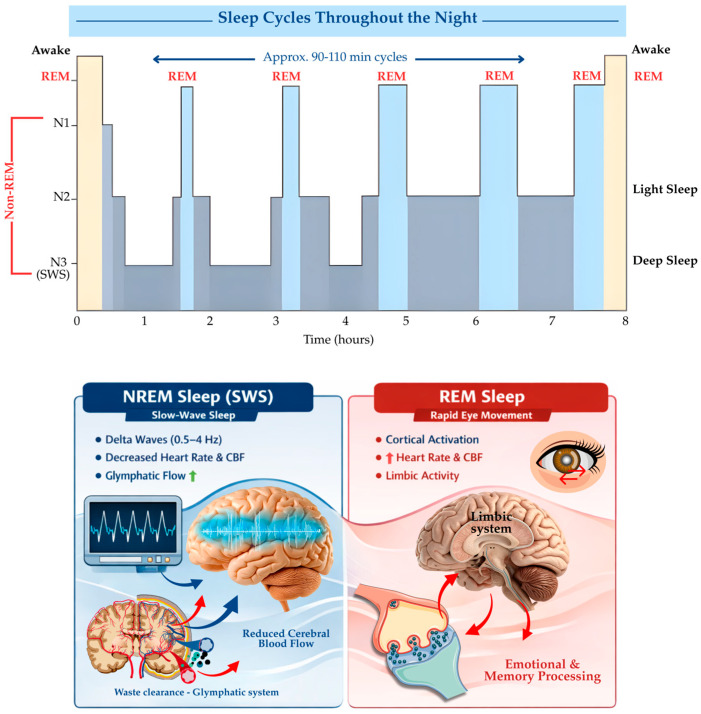
Sleep architecture–dependent regulation of neurovascular dynamics and glymphatic function. Normal human sleep consists of cyclic alternations between non–rapid eye movement (NREM) sleep and rapid eye movement (REM) sleep, recurring every ~90–110 min across the night ((**top**) panel). NREM sleep progresses from light sleep (N1–N2) to deep slow-wave sleep (N3/SWS), which is characterized by high-amplitude, low-frequency delta oscillations (0.5–4 Hz), parasympathetic predominance, and reductions in heart rate, cerebral blood flow (CBF), and cerebral blood volume (CBV). Despite reduced perfusion, preserved low-frequency vasomotor oscillations during SWS promote cerebrospinal fluid (CSF) movement along perivascular spaces, thereby enhancing glymphatic waste clearance and supporting synaptic homeostasis and cellular repair ((**left**) panel). In contrast, REM sleep is marked by cortical activation, sympathetic dominance, and increased variability in cardiovascular parameters, with region-specific elevations in CBF, particularly in limbic and paralimbic regions involved in emotional processing and memory integration; glymphatic influx during REM sleep appears more heterogeneous and relatively reduced compared with SWS ((**right**) panel). (Green and red upward-pointing arrows indicate an increase or elevation in the respective parameter. The left- and right-pointing red arrows within the “eye” symbol represent horizontal eye movements).

Normal human sleep is a highly organized and dynamic physiological process consisting of two principal states: NREM sleep and rapid eye movement (REM) sleep. These states alternate cyclically throughout the night in approximately 90–110 min intervals and serve complementary restorative and regulatory functions for the brain and peripheral organs (Figure 1) [21]. The architecture and continuity of these cycles are critical for maintaining neurovascular, metabolic, and cognitive homeostasis, with increasing evidence indicating that disruptions in normal sleep physiology can exert profound effects on cerebral perfusion and vascular integrity [22].

NREM sleep is subdivided into stages N1–N3, with stage N3, also referred to as slow-wave sleep (SWS), representing the deepest and most restorative phase. SWS is characterized by high-amplitude, low-frequency delta oscillations (0.5–4 Hz) and a marked predominance of parasympathetic activity [23]. This autonomic shift is accompanied by reductions in heart rate, systemic blood pressure, and cerebral metabolic demand, with studies reporting a 5–28% decrease in heart rate and regional brain perfusion during SWS [23]. These physiological changes create an energetically favorable state for synaptic downscaling, memory consolidation, and cellular repair processes, while simultaneously minimizing oxidative stress and excitotoxic burden [24,25].

From a cerebrovascular perspective, neuroimaging and near-infrared spectroscopy (NIRS) studies have demonstrated that SWS is associated with reduced cerebral blood flow (CBF) and cerebral blood volume (CBV) yet preserved low-frequency vasomotor oscillations [26]. This unique vascular profile allows for stable perfusion at reduced metabolic cost, thereby supporting neurovascular efficiency. Importantly, these oscillatory hemodynamic patterns are thought to enhance CSF movement within perivascular spaces, facilitating metabolic waste clearance through the glymphatic system (Figure 1) [7,27]. Thus, SWS represents a critical window during which sleep physiology, vascular dynamics, and glymphatic function converge.

In contrast, REM sleep is characterized by cortical activation, rapid eye movements, and a shift toward sympathetic dominance, accompanied by increased variability in heart rate and blood pressure [28]. REM sleep is associated with region-specific increases in CBF, particularly within limbic and paralimbic structures such as the amygdala and hippocampus, supporting emotional regulation and memory integration [29,30]. Glymphatic fluid dynamics during REM sleep appear more heterogeneous and stage-dependent, with evidence suggesting reduced CSF influx compared to SWS but preserved localized solute diffusion [31,32].

Interestingly, both animal and human studies consistently demonstrate that intact NREM–REM cycling, and particularly the preservation of SWS, is essential for optimal glymphatic clearance and neurovascular health [16,33]. Reductions in SWS and altered sleep architecture have been observed in aging, stroke, neurodegenerative disorders, and sleep-related breathing disorders, conditions that also exhibit a high burden of cerebral small vessel pathology [11,34]. These observations suggest that disturbances in normal sleep physiology may compromise vascular regulation and metabolic waste clearance, thereby increasing vulnerability to glymphatic dysfunction and cerebral small vessel disease.

### 2.2. Sleep Fragmentation (SF)

SF refers to the repetitive disruption of normal sleep continuity, characterized by frequent arousals that prevent the maintenance of consolidated NREM and REM sleep cycles, often occurring without full cortical awakenings [35]. Unlike total sleep deprivation, SF selectively impairs sleep architecture, disproportionately reducing SWS and destabilizing NREM–REM transitions. Clinically, SF arises from diverse etiologies, including obstructive sleep apnea (OSA), insomnia, aging, neurodegenerative diseases, circadian rhythm disturbances, and environmental stressors such as nocturnal noise and artificial light exposure [36].

From a vascular perspective, in OSA, SF co-occurs with intermittent hypoxia, hypercapnia, sympathetic surges, blood pressure variability, and oxidative stress [37,38]. These events are accompanied by acute surges in blood pressure, heart rate variability, and intrathoracic pressure, which together impose mechanical and oxidative stress on the cerebral microvasculature. Over time, this nocturnal hemodynamic instability contributes to endothelial dysfunction, arterial stiffening, impaired cerebral autoregulation, and BBB disruption, pathophysiological processes central to the development and progression of CSVD [26]. These physiological stressors likely contribute as much as, or more than, SF per se to vascular injury and CSVD risk. Accordingly, OSA should be viewed as a multifactorial vascular stress model rather than a pure sleep fragmentation phenotype.

Furthermore, SF per se has been shown to alter cerebrovascular tone and metabolic regulation even in the absence of hypoxia. Clinical studies demonstrate that recurrent arousals induce fluctuations in CBF and vascular pulsatility, impair nitric oxide (NO) bioavailability, and promote oxidative stress within the neurovascular unit [39]. These changes reduce the capacity of small cerebral vessels to respond appropriately to metabolic demands, thereby exacerbating perfusion heterogeneity and promoting white matter vulnerability [40]. Importantly, SF is increasingly recognized as a potential contributor to chronic low-grade inflammation, insulin resistance, and dyslipidemia, systemic metabolic disturbances [41,42] that may further compromise vascular health and are highly relevant to CSVD pathogenesis from a nutritional standpoint.

Moreover, objective quantification of SF relies on parameters such as the arousal index, sleep efficiency, sleep latency, and the relative duration of N3 and REM sleep [43,44]. These metrics are most accurately assessed using polysomnography, with complementary data provided by actigraphy and piezoelectric sleep monitoring systems in longitudinal and population-based studies [45]. Data from pre-clinical and clinical studies reported that elevated arousal index values and reduced sleep efficiency consistently reflect increased disruption of restorative sleep stages and have been associated with cognitive impairment, autonomic dysregulation, endothelial dysfunction, and markers of cerebrovascular injury, including WMHs burden and ePVS [46,47].

In addition, subjective assessments, such as the Pittsburgh Sleep Quality Index (PSQI) and Epworth Sleepiness Scale, provide valuable insights into perceived sleep quality and daytime dysfunction but lack the temporal resolution necessary to capture micro-arousals and nocturnal vascular stressors [48]. Consequently, the integration of objective sleep metrics with neuroimaging and vascular biomarkers is increasingly advocated to elucidate how SF drives microvascular injury and glymphatic dysfunction. Collectively, normal sleep architecture provides the physiological conditions required for efficient cerebrovascular regulation and metabolic waste clearance. In contrast, SF disrupts these coordinated neurovascular rhythms, creating a permissive environment for glymphatic failure. The following section, therefore, focuses on the structural and molecular organization of the glymphatic system and how sleep disruption destabilizes this clearance pathway.

### 2.3. SF and Brain Health

As discussed, SF exerts widespread effects on brain health by disrupting cerebral hemodynamics, autonomic regulation, and endothelial integrity. Clinical neuroimaging and physiological studies demonstrate that SF destabilizes CBF and tissue oxygenation, creating a fluctuating vascular environment that challenges cerebrovascular homeostasis [49,50]. Recurrent arousals are associated with persistent sympathetic activation, attenuation of normal nocturnal blood pressure dip, and increased nighttime blood pressure variability, phenomena that are strongly linked to hypertension and accelerated vascular aging [37]. In the cerebral microvasculature, these hemodynamic stresses impair endothelial function, reduce NO bioavailability, and promote oxidative stress, collectively compromising the ability of small vessels to maintain autoregulatory control [51].

At the structural level, endothelial dysfunction and tight junction disruption within cerebral vessels contribute to BBB permeability and perivascular leakage. These changes are closely associated with hallmark neuroimaging features of CSVD, including WMHs and ePVS [52,53]. Mechanistically, frequent micro-arousals trigger acute surges in heart rate and blood pressure, producing repetitive mechanical strain on fragile penetrating arterioles and capillaries [54]. Over time, this nocturnal vascular instability impairs cerebrovascular autoregulation, promotes microvascular rarefaction, and increases white matter susceptibility to hypoperfusion-related injury [55]. Beyond vascular stress, SF has been suggested to disrupt glymphatic clearance, further exacerbating brain vulnerability. The loss of consolidated NREM sleep, particularly SWS, may reduce CSF–ISF exchange and impair the removal of metabolic waste products, including amyloid-β, tau, and inflammatory mediators. Pre-clinical studies consistently demonstrate that SF diminishes glymphatic efficiency, leading to waste accumulation, neuroinflammation, and perivascular dysfunction [56,57]. These effects have been proposed to be compounded by altered vascular pulsatility and aberrant aquaporin-4 (AQP4) polarization, linking sleep disruption directly to impaired clearance mechanisms [58].

Furthermore, SF has been shown to negatively impact cognitive and metabolic brain function. Clinical and experimental studies report associations between SF and deficits in memory consolidation, executive function, and attentional control [37,56,59]. In rodent models, chronic SF increases cerebral glucose utilization, dysregulates hypothalamic–pituitary–adrenal (HPA) axis activity, and induces pro-inflammatory signaling within hippocampal and cortical regions, pathophysiological patterns that overlap with those observed in neurodegenerative and vascular cognitive disorders [60,61]. These findings underscore the tight coupling between sleep integrity, metabolic regulation, and neurovascular health.

**Figure 2:**
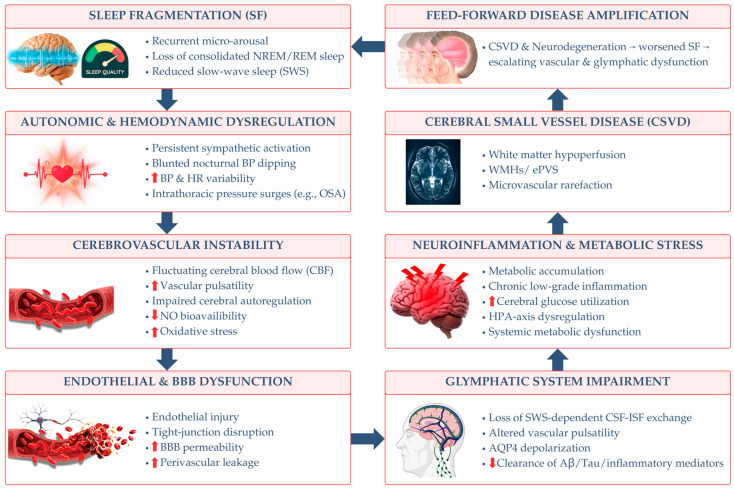
Conceptual framework illustrating sleep fragmentation (SF)–induced neuroglial dysregulation and glymphatic impairment at the cellular and molecular levels, highlighting astrocytic aquaporin 4 (AQP4) polarity, neurovascular coupling, and inflammatory signaling. SF disrupts autonomic regulation and cerebral hemodynamics, leading to cerebrovascular instability, endothelial and blood–brain barrier (BBB) dysfunction, and the development of cerebral small vessel disease (CSVD). Concomitantly, loss of slow-wave sleep (SWS) impairs glymphatic clearance through altered vascular pulsatility and AQP4 depolarization, promoting neuroinflammation, metabolic stress, and cognitive impairment. These processes form a feed-forward loop in which CSVD and neurodegeneration further exacerbate SF and brain injury. (Upward- and downward-pointing arrows indicate an increase/elevation and a decrease/reduction in the respective parameter).

Collectively, the architecture and continuity of sleep play a critical role in preserving cerebrovascular stability and supporting glymphatic function. Deep NREM sleep facilitates CSF–dependent waste clearance and maintains endothelial and autonomic balance, while REM sleep contributes to region-specific perfusion and metabolic integration [62]. Persistent SF, whether driven by intrinsic disorders or extrinsic stressors, disrupts these coordinated processes, resulting in heightened sympathetic activity, endothelial stress, impaired glymphatic clearance, and progressive cognitive dysfunction. Within the context of CSVD, SF may act both as an initiating insult and a disease-amplifying mechanism, linking vascular dysregulation, glymphatic failure, and white matter injury. These insights reinforce the concept of a sleep–glymphatic–vascular continuum and highlight the importance of integrating sleep metrics into CSVD models, while providing a mechanistic rationale for exploring neurovascular protective strategies, including nutraceutical interventions such as natural vitamins and minerals. Figure 2 summarizes the core mechanistic framework linking sleep fragmentation to glymphatic dysfunction via neuroglial dysregulation, emphasizing astrocytic AQP4 depolarization, impaired neurovascular coupling, and inflammatory amplification loops discussed in this section.

## 3. The Glymphatic System: Structure and Function

### 3.1. Anatomical and Molecular Components

**Figure 3:**
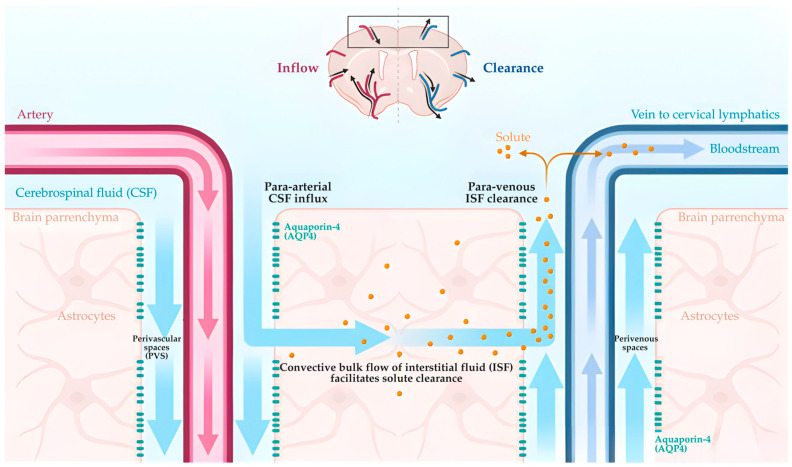
Schematic organization of the glymphatic system. Cerebrospinal fluid (CSF) enters the brain along periarterial spaces surrounding pial and penetrating arteries, driven primarily by arterial pulsatility. CSF exchanges convectively with interstitial fluid (ISF) through astrocytic endfeet enriched in polarized aquaporin-4 (AQP4), facilitating the clearance of metabolic waste products, including amyloid-β, tau, lactate, and α-synuclein. Interstitial solutes are subsequently transported toward perivenous spaces and drained via meningeal lymphatic vessels to deep cervical lymph nodes. Glymphatic function is enhanced during non-rapid eye movement (NREM) slow-wave sleep through increased arterial pulsatility and expansion of the interstitial space, whereas aging, sleep fragmentation, cerebrovascular small vessel disease (CSVD), blood–brain barrier (BBB) dysfunction, and AQP4 depolarization impair clearance efficiency, promoting protein aggregation, neuroinflammation, and neurodegeneration. (Blue and gold arrows indicate the movement of fluid and solutes).

The glymphatic system is a specialized perivascular clearance network that facilitates the removal of interstitial solutes from the brain and plays a fundamental role in maintaining cerebral metabolic and vascular homeostasis [63]. Unlike peripheral lymphatic systems, glymphatic transport relies on CSF movement along vascular pathways. CSF enters the brain parenchyma via para-arterial spaces surrounding penetrating arterioles, mixes with ISF, and exits along perivenous drainage routes [15]. This directional exchange supports the clearance of metabolic waste products, neurotoxic proteins, and inflammatory mediators. A central molecular determinant of glymphatic efficiency is AQP4, a water channel abundantly expressed in astrocytic end feet that ensheathe cerebral microvessels [64]. The polarized localization of AQP4 at perivascular astrocytic membranes enables low-resistance, directional CSF–ISF flux, thereby facilitating convective solute transport through the brain parenchyma (Figure 3). Loss of AQP4 polarization disrupts this organized fluid movement, leading to impaired clearance and perivascular fluid stagnation [64].

Perivascular spaces, historically referred to as Virchow–Robin spaces, constitute the anatomical conduits through which glymphatic flow occurs. These fluid-filled compartments accompany arterioles, capillaries, and venules and are structurally and functionally integrated within the neuro-glia-vascular unit (NGVU), comprising endothelial cells, pericytes, astrocytes, microglia, and extracellular matrix components [65]. Endothelial cells and pericytes regulate vascular tone, BBB permeability, and pulsatile forces that drive CSF movement, while microglia modulate inflammatory signaling that can secondarily influence astrocytic and vascular function [66,67]. Together, these cellular interactions underscore the intimate coupling between glymphatic transport and cerebrovascular integrity. In CSVD, pathological alterations in these anatomical and molecular components are frequently observed, whereby ePVS, endothelial dysfunction, BBB breakdown, and reduced AQP4 polarization are consistently reported across aging and vascular risk populations [68,69,70]. These changes impair perivascular fluid dynamics and promote the accumulation of neurotoxic proteins, reinforcing glymphatic dysfunction as a mechanistic interface between vascular pathology and neurodegenerative processes. Importantly, although ePVS is often interpreted as reflecting impaired perivascular clearance, ePVS enlargement is multifactorial and can reflect aging, hypertension, small vessel wall pathology, BBB leakage, altered interstitial fluid dynamics, and neuroinflammation. Thus, ePVS should be considered a non-specific structural marker of perivascular pathology rather than a direct signature of glymphatic failure.

### 3.2. Sleep-Dependent Glymphatic Function

Glymphatic transport is strongly regulated by sleep–wake states, with converging evidence from experimental and clinical studies demonstrating maximal clearance efficiency during NREM-SWS. In animal models, CSF influx into the brain increases substantially during sleep compared with wakefulness, coinciding with an expansion of ISF that reduces hydraulic resistance and enhances convective ISF flow toward perivenous drainage pathways [33]. This physiological state supports the efficient elimination of metabolic waste products, including amyloid-β, tau, and oxidative metabolites [71].

Additionally, noradrenergic signaling plays a critical regulatory role in this process, whereby during sleep, particularly SWS, reduced noradrenergic tone promotes astrocytic relaxation and extracellular space expansion, thereby facilitating CSF–ISF exchange [72,73]. In contrast, wakefulness or disrupted sleep is associated with elevated noradrenergic activity, contraction of perivascular spaces, reduced CSF influx, and impaired glymphatic efficiency [73]. Experimental SF further exacerbates these effects by inducing astrocytic reactivity and AQP4 mislocalization, thereby compromising the structural basis for directional fluid transport [74].

In humans, direct visualization of glymphatic flow remains challenging; however, advanced diffusion-based neuroimaging techniques have enabled indirect assessment of perivascular fluid dynamics. Diffusion tensor imaging–analysis along the perivascular space (DTI-ALPS) provides an indirect proxy of perivascular water diffusivity and does not directly quantify CSF–ISF flux. ALPS metrics are influenced by white matter microstructure, vascular pulsatility, tissue atrophy, diffusion anisotropy, scanner parameters, and processing pipelines [75]. Moreover, reduced DTI-ALPS indices have been associated with poor sleep quality, aging, vascular risk factors, and imaging markers of CSVD, including WMHs and ePVS [68,76]. These findings support the translational relevance of sleep-dependent glymphatic dysfunction in human cerebrovascular disease.

Collectively, evidence from animal models, neuroimaging, and clinical observations indicates that the glymphatic system operates as a sleep-dependent, astrocyte-mediated clearance pathway that is tightly coupled to cerebral vascular function. Disruption of sleep architecture, through fragmentation, loss of SWS, or altered noradrenergic signaling, impairs glymphatic transport and promotes metabolic waste accumulation. In the context of CSVD, structural abnormalities such as ePVS and reduced AQP4 polarization synergize with vascular dysfunction to further compromise clearance mechanisms, thereby accelerating neurodegeneration and cognitive decline. This synthesis reinforces a tripartite relationship between sleep, glymphatic function, and vascular health, and provides a mechanistic foundation for exploring nutritional and vascular-protective interventions, including T3, aimed at preserving astrocytic and endothelial integrity.

### 3.3. SF as a Driver of Glymphatic Dysfunction

Building on the sleep-dependent nature of glymphatic transport, SF is suggested to represent a potent upstream perturbation that directly disrupts the temporal and physiological conditions required for efficient CSF–ISF exchange. Unlike total sleep deprivation, SF may selectively erode the continuity and depth of NREM-SWS, resulting in repeated transitions toward wake-like neurochemical and autonomic states [77,78]. These frequent arousals reinstate noradrenergic signaling and destabilize cerebrovascular pulsatility, thereby interrupting the coordinated neuronal–vascular oscillations that normally drive periarterial CSF influx and solute clearance [73]. As a consequence, glymphatic transport becomes inefficient and intermittent, favoring progressive accumulation of metabolic waste rather than sustained clearance.

At the astrocytic–vascular interface, SF induces maladaptive structural and functional remodeling that further amplifies glymphatic failure. Recurrent sleep disruption has been shown to alter astrocytic end foot organization, leading to depolarization and mislocalization of AQP4 water channels from perivascular membranes [79]. Rather than reducing clearance capacity, AQP4 depolarization fundamentally alters fluid directionality within perivascular pathways, promoting CSF stagnation and impaired interstitial drainage. These changes create a permissive microenvironment for oxidative stress, chronic neuroinflammation, and endothelial injury, processes that are central to CSVD pathophysiology and are exacerbated by concomitant vascular risk factors [80].

The downstream structural consequences of SF-induced glymphatic dysfunction are increasingly captured by neuroimaging markers, particularly ePVS. While perivascular spaces normally serve as functional conduits for CSF transport, persistent impairment of glymphatic flow leads to their dilation and loss of clearance efficiency [81]. Importantly, ePVS burden shows consistent associations with SF severity, arousal frequency, aging, and vascular comorbidities, and has been associated with WMH burden, BBB permeability markers, and cognitive performance across observational cohorts, although reported effect sizes are modest to moderate and vary across populations and methodologies [82]. In this context, ePVS should be considered as non-specific structural markers of perivascular pathology that are compatible with impaired perivascular clearance but are also influenced by aging, vascular risk factors, vessel wall pathology, BBB dysfunction, and neuroinflammation. Accordingly, ePVS cannot be interpreted as a direct or specific signature of sleep-driven glymphatic failure.

Functional neuroimaging has strengthened the biological plausibility of a link between SF, perivascular fluid dynamics, and glymphatic impairment, although current evidence remains correlational and indirect. Despite current limitations, DTI-ALPS provides an indirect, yet sensitive measure of water diffusivity aligned with perivascular pathways and has emerged as a valuable in vivo proxy for glymphatic activity [68,75,83]. However, it does not directly quantify CSF–ISF flux and is influenced by white matter microstructure, vascular pulsatility, tissue atrophy, and methodological factors. Associations between elevated arousal indices, reduced SWS, and lower ALPS values in humans are consistent with, but do not establish, a mechanistic continuum in which sleep fragmentation may contribute to glymphatic vulnerability [75,83]. Directionality and causality require confirmation in longitudinal and interventional studies. Reduced DTI-ALPS indices have been consistently reported in individuals with fragmented sleep, sleep disorders, and vascular cognitive impairment, and show inverse relationships with ePVS burden and white matter injury [84,85]. Notably, associations between elevated arousal indices, reduced SWS, and lower ALPS values in human studies are consistent with a mechanistic continuum in which SF may contribute to functional glymphatic vulnerability; however, directionality and temporal precedence remain to be established in longitudinal and interventional studies.

Together, evidence from experimental models supports SF as a causal driver of glymphatic dysfunction, whereas human neuroimaging studies provide converging associative evidence consistent with this mechanistic framework rather than direct proof of causality. Through coordinated effects on sleep architecture, astrocytic water channel organization, and perivascular fluid dynamics, SF promotes metabolic waste accumulation, vascular stress, and neuroinflammation in pre-clinical models, whereas in humans, SF is associated with imaging and biomarker signatures consistent with these processes, thereby supporting a link to CSVD vulnerability and cognitive decline. Integrating functional measures such as DTI-ALPS with structural markers, including ePVS, offers a powerful framework for quantifying sleep-related glymphatic impairment in vivo and provides a mechanistic bridge linking sleep quality to small vessel pathology. These insights also underscore the therapeutic potential of interventions aimed at preserving sleep continuity and stabilizing astrocytic–vascular coupling as strategies to restore glymphatic function and mitigate CSVD progression. Thus, disruption of sleep-dependent glymphatic clearance establishes a mechanistic bridge between SF and microvascular vulnerability. The downstream consequences of impaired perivascular clearance on small vessel pathology and white matter injury are discussed in the subsequent section on CSVD.

Importantly, current human evidence linking SF, glymphatic dysfunction, and CSVD remains largely indirect and associative. Imaging markers such as DTI-ALPS and ePVS provide valuable in vivo windows into perivascular fluid dynamics and perivascular remodeling, but do not directly quantify CSF–ISF flux and are influenced by multiple vascular and structural factors. Accordingly, the sleep–glymphatic–vascular continuum presented here should be interpreted as a mechanistic framework grounded in strong preclinical causality and emerging human associations, rather than as a proven causal chain in humans. Longitudinal and interventional studies integrating objective sleep phenotyping with standardized glymphatic imaging will be required to establish directionality and clinical causality.

## 4. Glymphatic Dysfunction and CSVD

Building on the preceding sections, CSVD can be conceptualized as a downstream manifestation of chronic sleep–glymphatic uncoupling superimposed on vascular aging and metabolic stress.

### 4.1. CSVD Pathological Hallmarks in the Context of Impaired Glymphatic Clearance

CSVD is characterized by a constellation of structural brain lesions that reflect chronic microvascular injury and progressive failure of tissue homeostasis. While these hallmarks have traditionally been attributed to hemodynamic stress and endothelial pathology, growing evidence indicates that impaired glymphatic clearance critically shapes their development and spatial distribution. WMHs represent the most prevalent imaging manifestation of CSVD and arise from a combination of chronic hypoperfusion, BBB leakage, and ISF dysregulation [86,87]. In the setting of glymphatic dysfunction, inefficient removal of metabolic byproducts, excess ISF, and inflammatory mediators leads to prolonged white matter exposure to toxic solutes [88]. This clearance failure exacerbates oligodendrocyte injury, myelin rarefaction, and axonal degeneration, thereby amplifying the WMHs burden beyond what would be expected from vascular insufficiency alone. Importantly, WMHs tend to localize along long penetrating arteries and periventricular regions [89], areas highly dependent on intact perivascular fluid exchange, supporting a role for disrupted glymphatic flow in their pathogenesis.

Moreover, lacunes and CMBs further reflect the convergence of vascular fragility and impaired perivascular homeostasis. Lacunar infarcts result from occlusion or collapse of deep perforating arterioles [90], while microbleeds signify focal rupture of structurally weakened microvessels [91]. Glymphatic failure contributes to both lesion types by promoting perivascular accumulation of reactive oxygen species (ROS), hemoglobin breakdown products, and misfolded proteins that exert direct toxicity on vascular smooth muscle cells and pericytes [86]. Over time, this toxic milieu accelerates vessel wall degeneration, impairs autoregulatory capacity, and increases susceptibility to ischemic and hemorrhagic injury. Furthermore, ePVS occupies a central position at the interface between glymphatic dysfunction and CSVD pathology. Beyond serving as passive imaging markers, ePVS reflect structural remodeling of perivascular compartments in response to chronic clearance failure [18]. Persistent stagnation of CSF within perivascular pathways leads to their dilation, altered compliance, and loss of efficient solute transport. Hence, the strong associations between ePVS burden, WMHs, cognitive impairment, and BBB disruption suggest that ePVS represent a structural signature of long-standing glymphatic–vascular uncoupling.

### 4.2. Mechanistic Overlap Between Glymphatic Failure and CSVD Progression

The pathological overlap between glymphatic dysfunction and CSVD is underpinned by shared mechanisms that reinforce one another in a self-perpetuating cycle of vascular and interstitial injury. Endothelial dysfunction represents a pivotal convergence point. Accumulation of metabolic waste products, oxidized lipids, and inflammatory cytokines within perivascular spaces exerts sustained stress on endothelial cells, impairing NO bioavailability and disrupting vasomotor signaling [92,93]. This endothelial impairment reduces arterial pulsatility, a key driver of glymphatic transport, thereby further diminishing clearance efficiency. As vascular reactivity declines, perivascular flow becomes increasingly stagnant, amplifying solute accumulation and vascular toxicity [94].

Additionally, BBB breakdown both results from and contributes to impaired glymphatic function, whereby chronic exposure to inflammatory mediators and oxidative stress weakens tight junction integrity, increasing BBB permeability [53]. Meanwhile, plasma-derived proteins such as fibrinogen and albumin then enter the perivascular and interstitial compartments, where they exacerbate astrocytic activation and microglial priming [95,96]. These changes alter astrocytic end foot structure and AQP4 organization, further compromising perivascular fluid dynamics and reinforcing glymphatic failure. Moreover, chronic neuroinflammation constitutes another critical link between impaired clearance and small vessel pathology; for example, inefficient removal of damage-associated molecular patterns (DAMPs), amyloidogenic peptides, and lipid peroxidation products sustains microglial activation and astrocytic reactivity [97]. Rather than resolving injury, this low-grade inflammatory state promotes extracellular matrix remodeling, perivascular fibrosis, and progressive vessel stiffening [98]. These alterations reduce vessel compliance and impair the biomechanical forces required for effective CSF–ISF exchange.

Collectively, impaired waste clearance transforms the cerebral microenvironment into one that is intrinsically hostile to vascular integrity. Accumulating neurotoxic solutes and inflammatory mediators directly injure endothelial cells, pericytes, and vascular smooth muscle cells, leading to vessel wall thickening, lumen narrowing, and increased rupture risk. In this framework, glymphatic dysfunction is not merely a consequence of CSVD but an active contributor that accelerates microvascular degeneration, lesion accumulation, and cognitive decline. Taken together, glymphatic failure and CSVD should be viewed as interdependent pathologies linked by disrupted perivascular homeostasis, endothelial injury, and chronic inflammation. This integrated perspective provides a mechanistic foundation for targeting clearance pathways, astrocytic–vascular coupling, and metabolic toxicity as complementary strategies to traditional vascular risk modification in CSVD management.

### 4.3. The Vicious Cycle: SF, Glymphatic Failure, and CSVD

Emerging evidence supports a bidirectional and self-reinforcing relationship between SF, glymphatic dysfunction, and CSVD, forming a pathological feedback loop that accelerates brain aging and cognitive decline. Rather than acting in isolation, these processes interact dynamically, with impairment in any one domain propagating dysfunction across the others. Pre-clinical studies have shown that SF acts as a primary destabilizing force by disrupting the temporal and physiological conditions required for effective perivascular fluid transport [99]. Loss of consolidated SWS reduces cerebrovascular pulsatility, alters autonomic balance, and limits the rhythmic arterial wall movements that drive glymphatic flow [100]. As glymphatic efficiency declines, metabolic waste products, inflammatory mediators, and excess interstitial fluid accumulate within perivascular compartments, directly injuring small vessels and surrounding glial structures.

Progressive CSVD, in turn, feeds back to further impair sleep and glymphatic function. Structural vessel wall changes, including arteriolosclerosis, increased vascular stiffness, and reduced compliance of penetrating arteries, attenuate pulsatile forces essential for cerebrospinal fluid propulsion [101]. These vascular alterations not only compromise glymphatic transport but also disrupt neurovascular coupling and regional perfusion, increasing the likelihood of sleep instability, nocturnal arousals, and impaired sleep continuity [12,22]. Thus, CSVD transforms from an outcome of glymphatic failure into a driver of its persistence.

Furthermore, vascular stiffness occupies a central mechanistic position within this cycle. Age-related and disease-related increases in arterial rigidity blunt the mechanical forces required for perivascular exchange, rendering the glymphatic system increasingly dependent on optimal sleep architecture [102,103]. When sleep becomes fragmented, this dependence is unmasked, resulting in disproportionate clearance failure even in the absence of overt vascular occlusion. This interaction helps explain why sleep disturbances exert particularly strong effects on CSVD progression in older adults. In addition, aging and metabolic comorbidities further amplify this triadic interaction, whereby conditions such as hypertension, diabetes, dyslipidemia, and obesity exacerbate endothelial dysfunction, promote low-grade systemic inflammation, and impair astrocytic–vascular signaling [104]. Recent experimental studies directly link modulation of lipid metabolism and vascular inflammation to post-stroke cognitive outcomes, providing causal support for vascular–metabolic–cognitive coupling [105,106]. These findings strengthen the conceptual bridge between metabolic dysregulation, small vessel pathology, and cognitive impairment. Overall, these factors accelerate both vascular stiffening and glymphatic inefficiency, lowering the threshold at which sleep fragmentation precipitates pathological changes. Consequently, individuals with metabolic risk profiles may enter this vicious cycle earlier and progress more rapidly toward structural brain injury.

**Figure 4:**
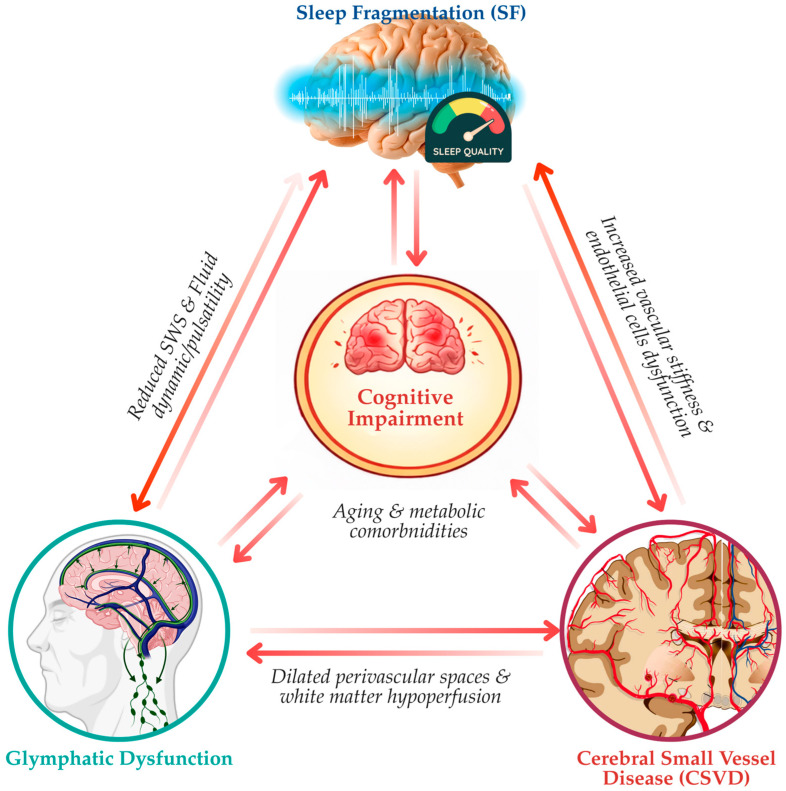
Triangular feedback loop illustrating the bidirectional interactions between sleep fragmentation (SF), glymphatic dysfunction, and cerebral small vessel disease (CSVD). SF reduces slow-wave sleep (SWS) and cerebrovascular pulsatility, impairing glymphatic clearance. Glymphatic failure promotes toxic accumulation and perivascular remodeling, accelerating CSVD. Progressive CSVD increases vascular stiffness and disrupts neurovascular coupling, further destabilizing sleep architecture. Aging and metabolic comorbidities act as amplifiers, while cognitive impairment represents a downstream clinical consequence.

At the clinical level, the convergence of SF, glymphatic failure, and CSVD provides a mechanistic substrate for cognitive impairment and dementia. Accumulation of neurotoxic proteins, chronic white matter injury, and persistent neuroinflammation disrupt large-scale brain networks involved in attention, executive function, and memory [107]. Importantly, cognitive decline may further worsen sleep quality, reinforce the cycle, and contribute to the heterogeneity and progression of vascular and mixed dementias [108]. Hence, conceptualizing SF, glymphatic dysfunction, and CSVD as components of a unified feedback loop reframes CSVD from a purely vascular disorder to a systems-level failure of clearance, vascular biomechanics, and sleep regulation. This framework highlights the potential of multimodal interventions targeting sleep continuity, vascular compliance, and astrocytic–perivascular integrity to interrupt disease progression. Building on the mechanistic model in Figure 2, Figure 4 extends this framework to the clinical and neuroimaging domain, illustrating how chronic glymphatic impairment manifests as CSVD phenotypes, including ePVS enlargement, white matter injury, and BBB disruption.

## 5. Tocotrienols (T3): Biological Properties Relevant to Neurovascular Protection

### 5.1. T3 Isoforms, Bioavailability, and Nutraceutical Context

**Figure 5:**
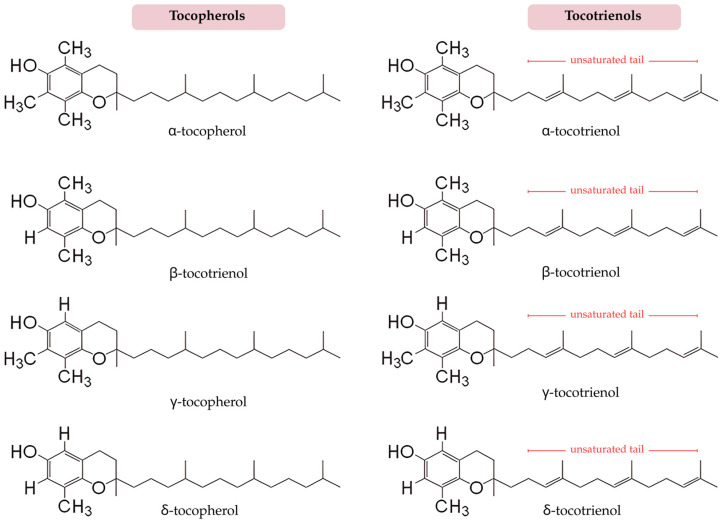
Comparison of the chemical structure of tocotrienols (T3) versus tocopherols. Both T3 and tocopherols comprise four isoforms (α-, β-, γ-, and δ-) defined by the number and position of methyl groups on the chromanol ring. T3s are distinguished by an unsaturated isoprenoid side chain containing three double bonds.

Vitamin E is a collective term encompassing two structurally distinct families: tocopherols and tocotrienols (T3), each comprising four isoforms (α-, β-, γ-, and δ-) defined by the number and position of methyl groups on the chromanol ring (Figure 5). While tocopherols possess a saturated phytyl tail, T3s are distinguished by an unsaturated isoprenoid side chain containing three double bonds, a feature that confers unique physicochemical and biological properties [109]. This unsaturated side chain can enhances membrane fluidity, lateral mobility within lipid bilayers, and may facilitate penetration across lipid-rich biological barriers, including the BBB in preclinical models [110]. However, limited preclinical data suggest that certain T3 isoforms can be detected in brain tissue under specific dosing and formulation conditions; however, clinical brain pharmacokinetic data remain sparse, and brain exposure is likely influenced by dose, formulation, lipid co-ingestion, and interindividual variability. Hence, claims regarding BBB penetration should be interpreted cautiously in the absence of robust human brain pharmacokinetic studies. Consequently, T3 demonstrates differential tissue distribution compared with tocopherols, with preclinical studies suggesting higher accumulation in metabolically active and lipid-dense organs such as the liver, vascular endothelium, and, under specific conditions, the brain [111]. These properties are especially relevant within the context of CSVD, where endothelial integrity, astrocytic function, and perivascular signaling are compromised.

Among T3 isoforms, α-T3 has emerged as the most extensively studied and biologically potent in the central nervous system. Preclinical and clinical pharmacokinetic studies indicate that α-T3 achieves higher plasma stability and greater brain accumulation relative to γ- and δ-isoforms when administered either as isolated compounds or within tocotrienol-rich fractions (TRF) [19]. Importantly, α-T3 has been shown to exert biological effects at nanomolar concentrations, suggesting high functional bio-efficacy even under physiological exposure levels [19,112]. TRF, mostly derived from palm oil, provides a natural matrix of T3 (typically 70–80%) with smaller amounts of tocopherols, offering potential advantages in bioavailability, metabolic stability, and synergistic activity [113,114]. Evidence from animal models and human supplementation studies demonstrates that TRF can cross the BBB and accumulate within brain tissue, although penetration efficiency may vary depending on BBB integrity, vascular health, age, and metabolic status [115]. These variables are particularly pertinent in aging populations and individuals with CSVD, where BBB permeability and endothelial function are altered.

T3 are naturally occurring members of the vitamin E family derived primarily from palm oil, annatto seeds, rice bran oil, and certain cereal grains. However, habitual dietary intake of T3 varies widely across populations and is generally low in non–palm-oil-consuming regions, raising important translational considerations regarding the feasibility of achieving biologically meaningful exposure through diet alone [116,117]. Reported habitual intake in most populations is substantially lower than doses employed in experimental and interventional studies, which typically rely on concentrated TRF or annatto-derived supplements [118]. From a nutritional and translational perspective, the capacity of T3 particularly α-T3 to reach neurovascular and perivascular compartments supports their candidacy as modulators of oxidative stress, endothelial signaling, astrocytic homeostasis, and inflammatory tone. Although direct evidence linking T3 bioavailability to glymphatic modulation remains limited, their favorable brain distribution profile establishes a biological prerequisite for influencing astrocyte-mediated clearance pathways and vascular–glial interactions implicated in sleep-related glymphatic dysfunction. Collectively, the structural distinctiveness, pleiotropic molecular actions, and brain bioavailability of T3 isoforms provide a strong biochemical foundation for their exploration as nutritional neurovascular protectants, particularly within disease contexts characterized by sleep disruption, vascular stiffness, and impaired perivascular clearance.

Within a broader nutritional systems framework, T3 can be positioned alongside other nutraceuticals with emerging relevance to sleep regulation, vascular integrity, and neuroinflammation, including omega-3 fatty acids (vascular compliance and anti-inflammatory actions) [119], melatonin (circadian and sleep architecture modulation) [120], polyphenols such as resveratrol and flavonoids (endothelial protection and antioxidant signaling) [121], magnesium (sleep quality and autonomic regulation), and selected herbal adaptogens with sleep-modulatory potential [122]. While T3 offers distinctive pleiotropic actions on redox balance, endothelial biology, and mitochondrial function, comparative effectiveness and potential synergistic interactions among these nutraceuticals remain insufficiently characterized in the context of glymphatic function and CSVD risk. Long-term T3 supplementation appears feasible based on available safety data, with favorable tolerability profiles reported in cardiovascular and metabolic populations. Nonetheless, bioavailability is influenced by formulation, lipid co-ingestion, and interindividual variability in absorption and metabolism, underscoring the need for standardized formulations, dose–response evaluation, and pharmacokinetic optimization in future neurovascular and sleep-focused translational studies.

### 5.2. Antioxidant and Anti-Inflammatory Actions

Oxidative stress and chronic low-grade inflammation are central drivers of neurovascular dysfunction in CSVD and are increasingly implicated in SF–associated brain injury. The excessive generation of ROS disrupts endothelial homeostasis, compromises the BBB integrity, and promotes pathological protein modifications, including tau hyperphosphorylation and lipid peroxidation, thereby amplifying neurodegenerative and vascular injury cascades [123]. T3 exerts potent antioxidant effects that extend beyond classical radical scavenging. A key mechanism involves the activation of the nuclear factor erythroid 2–related factor 2 (Nrf2) signaling pathway, which upregulates endogenous antioxidant defenses, including superoxide dismutase (SOD), catalase (CAT), and glutathione peroxidase (GPx). Through this coordinated response, T3 has been proposed to reduce oxidative injury within endothelial cells, astrocytes, and neurons, thereby preserving cellular resilience under conditions of metabolic and vascular stress [124,125]. This mechanism is particularly relevant in SF, where intermittent hypoxia, sympathetic overactivity, and mitochondrial stress accelerate ROS generation.

In parallel, T3 exhibits pronounced anti-inflammatory activity by modulating key transcriptional regulators of innate immune signaling. TRF has been shown to inhibit nuclear factor kappa-light-chain-enhancer of activated B cells (NF-κB) activation, suppress downstream expression of pro-inflammatory mediators such as cyclooxygenase-2 (COX-2), inducible nitric oxide synthase (iNOS), and tumor necrosis factor-α (TNF-α), and attenuate microglial and astrocytic inflammatory responses [126,127]. These effects are particularly relevant in the context of glymphatic dysfunction, where impaired waste clearance fosters a pro-inflammatory perivascular microenvironment.

Experimental studies further demonstrate that TRF reduces lipid peroxidation and enhances astrocyte viability under excitotoxic and oxidative conditions, suggesting a protective effect on glial cells that are essential for perivascular homeostasis and AQP4-dependent fluid transport [128]. Clinically, T3 supplementation has been associated with improvements in systemic oxidative stress markers and inflammatory profiles, with emerging evidence suggesting potential benefits for cognitive performance and subjective sleep quality, although mechanistic human data remain limited [129]. Collectively, these antioxidant and anti-inflammatory actions position tocotrienols as modulators of the oxidative–inflammatory axis that links sleep fragmentation, glymphatic impairment, and microvascular injury.

### 5.3. Endothelial and BBB Protection

Endothelial dysfunction and BBB breakdown represent converging pathological processes in CSVD and play a critical role in amplifying glymphatic failure and sleep-related neurovascular injury. Oxidative stress and inflammation disrupt tight junction proteins, impair endothelial NO bioavailability, and alter vasomotor responsiveness, resulting in increased vascular permeability, impaired autoregulation, and reduced cerebral perfusion stability [100]. T3 has demonstrated protective effects on endothelial structure and function through multiple complementary mechanisms. By reducing oxidative stress and preserving NO signaling, T3 enhances endothelium-dependent vasodilation and mitigates vascular stiffness, factors that are essential for maintaining cerebrovascular pulsatility and perivascular fluid dynamics [130,131]. Restoration of endothelial redox balance may also indirectly support glymphatic transport, which depends on intact vascular–astrocytic coupling and arterial wall compliance.

Beyond redox modulation, TRF influences intracellular signaling pathways involved in endothelial survival and permeability regulation, including protein kinase C (PKC) and vascular endothelial growth factor (VEGF)–related signaling. These effects may contribute to stabilization of tight junction complexes and reduced leukocyte transmigration, thereby limiting BBB leakage and perivascular inflammation [132,133]. Importantly, TRF has been shown to cross the BBB via mechanisms such as endocytosis or transcytosis, suggesting direct access to neurovascular and glial targets [20]. Moreover, BBB integrity is a critical determinant of T3 delivery to the brain, and paradoxically, early BBB dysfunction in CSVD may initially enhance penetration while simultaneously exacerbating neurovascular vulnerability. Preclinical studies suggest that T3 supplementation can attenuate both vascular and neuronal damage in models of cerebrovascular and neurodegenerative pathology, supporting a role in preserving neurovascular unit integrity under stress conditions [134,135].

Taken together, the bioavailability, antioxidant and anti-inflammatory actions, and endothelial-protective properties of T3 converge to support a multilevel neurovascular protective profile. Within the framework of the sleep–glymphatic–vascular continuum, T3 may help disrupt the self-reinforcing cycle linking SF, impaired perivascular clearance, and CSVD progression by stabilizing endothelial function, maintaining BBB integrity, and reducing oxidative–inflammatory burden. While direct evidence for tocotrienol-mediated enhancement of glymphatic flux remains to be established, their mechanistic alignment with key upstream drivers of glymphatic failure warrants further targeted investigation in both experimental and clinical settings.

## 6. T3 and the Glymphatic–Sleep–Vascular Pathways

The convergence of SF, glymphatic dysfunction, and CSVD suggests that effective preventive strategies must operate across astrocytic, vascular, and inflammatory domains. T3, by virtue of its brain bioavailability and pleiotropic biological actions, may influence multiple nodes within this interconnected system. Although direct experimental evidence demonstrating T3-mediated enhancement of glymphatic flux is currently lacking, accumulating data support their capacity to modulate upstream biological determinants of glymphatic efficiency, including astrocytic redox balance, endothelial integrity, vascular compliance, neuroinflammation, and sleep-related neurovascular regulation. Accordingly, the proposed links between T3 and glymphatic function in this section should be interpreted as hypothesis-generating and mechanistically inferential, rather than as established causal relationships.

### 6.1. Potential Effects on Glymphatic Function

Efficient glymphatic transport critically depends on the polarized expression of AQP4 at astrocytic end feet surrounding cerebral vessels. This polarization is maintained by a specialized multiprotein anchoring complex composed of dystrophin (gene: *Dmd*), dystrophin-associated glycoprotein such as dystroglycan (gene: *Dag1*), and α-syntrophin (gene: *Snta1*), which collectively tether AQP4 to the perivascular astrocytic membrane. Dystroglycan serves as a transmembrane scaffold linking astrocytic end feet to the vascular basement membrane, while dystrophin connects this complex to the intracellular actin cytoskeleton, providing structural stability [136,137]. α-Syntrophin functions as a critical adaptor protein that directly interacts with the C-terminal PDZ-binding domain of AQP4, ensuring its precise localization and high-density clustering at perivascular domains. Disruption of any component of this dystrophin–glycoprotein complex leads to AQP4 mislocalization, reduced perivascular water permeability, and impaired CSF-ISF exchange, features consistently observed in aging, SF, and CSVD [138]. Evidence from genetic models further supports the importance of this anchoring complex, as α-syntrophin–deficient (*Snta1*^−^/^−^) and dystrophin-deficient (*Dmd*^−^/^−^) mice exhibit marked loss of perivascular AQP4 polarization, reduced CSF influx, impaired interstitial solute clearance, and enlargement of perivascular spaces, despite preserved total AQP4 expression, underscoring that spatial localization rather than channel abundance is critical for glymphatic efficiency [139].

Moreover, oxidative stress and chronic inflammation are key upstream drivers of AQP4 depolarization and perivascular dysfunction. Experimental studies demonstrate that excessive ROS and sustained exposure to pro-inflammatory cytokines, particularly TNF-α, IL-1β, and IL-6, suppress the expression and functional stability of *Dmd* and *Snta1*, disrupt actin cytoskeletal organization, and weaken astrocytic end foot attachment to the vascular basement membrane [140]. These molecular alterations destabilize the dystrophin–glycoprotein complex, promoting mislocalization of AQP4 away from perivascular domains and reducing perivascular water permeability, ultimately impairing CSF–ISF exchange and glymphatic clearance [141]. In parallel, oxidative injury to astrocytic mitochondria exacerbates calcium dysregulation and cytoskeletal remodeling, further accelerating AQP4 depolarization [142]. T3, through activation of Nrf2-dependent antioxidant pathways, is hypothesized to indirectly preserve AQP4 polarization by maintaining astrocytic redox balance, limiting mitochondrial dysfunction, and protecting the structural integrity of the dystrophin–glycoprotein anchoring complex [143]. By attenuating lipid peroxidation and ROS-driven inflammatory signaling within astrocytes and adjacent endothelial cells, T3 potentially reduces the molecular and structural stressors that precede AQP4 redistribution, thereby supporting perivascular fluid dynamics and glymphatic function under conditions of sleep fragmentation and vascular stress.

In addition, T3’s anti-inflammatory effects may further support glymphatic function by suppressing astrocyte reactivity (astrogliosis), a state associated with reduced AQP4 polarization and impaired perivascular clearance [144]. While T3 has not been directly shown to regulate AQP4 expression or polarization in vivo, their antioxidant and anti-inflammatory actions may indirectly preserve astrocytic end foot integrity and the dystrophin–glycoprotein anchoring complex, which are critical for perivascular AQP4 localization. This proposed mechanism remains speculative and requires targeted experimental validation using glymphatic imaging and astrocyte-specific molecular assays. Finally, at the structural level, preservation of endothelial function and vascular compliance by T3 may also facilitate glymphatic transport. Arterial pulsatility is a key driving force for CSF influx along periarterial spaces, and vascular stiffening in CSVD markedly reduces this propulsion [134,135]. Thus, T3-mediated improvements in endothelial nitric oxide bioavailability and vascular elasticity may therefore enhance the mechanical forces required for perivascular fluid movement.

### 6.2. T3 and Sleep Regulation

Sleep architecture, particularly the continuity and depth of SWS, plays a central role in regulating glymphatic clearance. SF increases sympathetic tone, elevates noradrenergic signaling, and suppresses the interstitial space expansion required for effective solute clearance [145]. These processes are closely linked to oxidative stress and neuroinflammation within sleep-regulating brain regions, including the hypothalamus, brainstem, and basal forebrain [100]. T3 may influence sleep regulation indirectly through its anti-inflammatory and antioxidant effects on neural circuits involved in sleep–wake control. Neuroinflammation within these regions is associated with impaired sleep continuity and reduced SWS, while antioxidant interventions have been shown to normalize sleep architecture in preclinical models [144,146]. Clinical studies reporting improved subjective sleep quality following T3 supplementation support this possibility, although objective polysomnographic data remain sparse.

Importantly, microglial activation occupies a central regulatory position at the intersection of sleep homeostasis, neuroinflammation, and glymphatic function. Under physiological conditions, microglia exhibit a highly dynamic surveillance phenotype that contributes to synaptic pruning, extracellular matrix remodeling, and the regulation of neuronal network activity involved in sleep–wake transitions [147]. SF disrupts this homeostatic state and promotes a shift toward a pro-inflammatory microglial phenotype, characterized by increased expression of activation markers such as Iba1, CD68, CD86, and inducible nitric oxide synthase (iNOS), alongside elevated production of pro-inflammatory cytokines including interleukin-1β (IL-1β), TNF-α, and IL-6 [148]. These mediators interfere with synaptic scaling and plasticity, perturb neuronal oscillatory activity critical for SWS, and reinforce sleep instability [149]. Beyond synaptic effects, sustained microglial inflammation exerts deleterious influences on astrocytic function and perivascular homeostasis. Pro-inflammatory microglia release NO, ROS, and matrix-modifying enzymes that disrupt astrocytic end feet integrity, reduce AQP4 polarization, and CSF-ISF exchange [7]. This inflammatory microenvironment fosters perivascular congestion and glymphatic inefficiency, linking microglial activation directly to impaired waste clearance and vascular stress.

Furthermore, recent work has refined the neuroinflammatory landscape beyond generalized ‘microglial activation,’ highlighting immune cell–specific dysfunction and context-dependent residency phenotypes that shape CNS injury and recovery trajectories [150,151]. These studies demonstrate that microglia, perivascular macrophages, and infiltrating myeloid populations adopt distinct transcriptional and functional states in response to vascular and metabolic stress, with differential implications for BBB integrity, perivascular remodeling, and tissue repair. Incorporating this cellular heterogeneity refines mechanistic interpretations linking sleep disruption, glymphatic impairment, and CSVD, as immune cell phenotypes may differentially modulate astrocyte–vascular coupling and perivascular clearance dynamics.

Interestingly, T3 has been shown to attenuate microglial activation by suppressing NF-κB–dependent inflammatory signaling, reducing the expression of iNOS and pro-inflammatory cytokines, and limiting oxidative stress within microglial cells [152]. Emerging evidence suggests that T3 may also bias microglial responses toward a more reparative and homeostatic phenotype, associated with increased expression of markers such as arginase-1 (Arg1), CD206, interleukin-10 (IL-10), and transforming growth factor-β (TGF-β) [153]. This shift may promote resolution of neuroinflammation, support astrocytic polarity, and preserve perivascular function. Thus, through modulation of microglial polarization, T3 may indirectly stabilize neuronal network activity and sleep architecture, reducing arousal frequency and sympathetic activation. In doing so, they may attenuate the neurovascular stress imposed by SF and help restore conditions favorable for glymphatic clearance.

Although direct causal links between T3-mediated microglial modulation and improved sleep or glymphatic function remain to be established, the convergence of anti-inflammatory, antioxidant, and neurovascular effects provides a compelling mechanistic framework for their potential role within the sleep–glymphatic–vascular continuum. Importantly, current evidence linking T3 supplementation to improvements in sleep architecture is limited to indirect or subjective measures, and no studies have yet demonstrated direct modulation of SWS or arousal indices using polysomnography; therefore, any sleep–glymphatic benefits of tocotrienols should be regarded as preliminary and hypothesis-driven.

### 6.3. Implications for CSVD Prevention and Progression

Within the context of CSVD, glymphatic failure, SF, and microvascular pathology operate as a tightly coupled, self-reinforcing system that accelerates white matter injury, cognitive decline, and progression toward vascular and mixed dementias [68,141]. Disrupted sleep architecture amplifies sympathetic tone and oxidative stress, impaired glymphatic clearance promotes perivascular congestion and toxic metabolite accumulation, and progressive vascular stiffening further destabilizes sleep and clearance mechanisms [68]. Interrupting this cycle at early or intermediate stages may therefore be critical for modifying disease trajectory. T3 may intervene at multiple mechanistic nodes within this pathogenic loop. By preserving endothelial function and stabilizing BBB integrity, T3 may limit plasma protein extravasation, leukocyte infiltration, and perivascular inflammation, processes that contribute to ePVS and white matter damage in CSVD [154]. Concurrently, by attenuating oxidative stress and suppressing chronic inflammatory signaling, T3 may help maintain astrocytic end foot integrity and AQP4 polarization, while promoting a more homeostatic microglial phenotype [152]. These effects collectively create a microenvironment more permissive to efficient glymphatic clearance.

**Figure 6:**
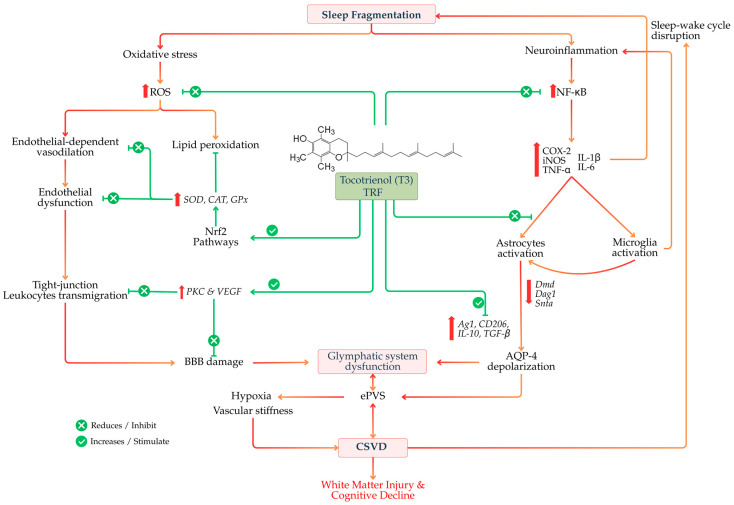
Systems pharmacology model depicting tocotrienol (T3)-centered network modulation of sleep–glymphatic–neurovascular pathways, highlighting therapeutic entry points and translational intervention opportunities. Sleep fragmentation (SF) induces oxidative stress and neuroinflammation, leading to endothelial dysfunction, blood–brain barrier (BBB) disruption, microglial and astrocytic activation, loss of aquaporin 4 (AQP4) polarization via destabilization of the Aqp4–Dmd–Dag1–Snta1 anchoring complex, and impaired glymphatic clearance, ultimately promoting enlarged perivascular spaces (ePVS), CSVD, and white matter injury with cognitive decline. T3 counteracts these processes by activating Nrf2-dependent antioxidant pathways, suppressing NF-κB–driven inflammatory signaling, preserving endothelial integrity and vascular compliance, biasing microglia toward a reparative phenotype, and stabilizing astrocytic AQP4 polarization, thereby supporting glymphatic function and mitigating CSVD progression.

In addition, modulation of neuroinflammatory pathways implicated in sleep instability suggests that T3 may indirectly support sleep continuity and SWS preservation, particularly in populations prone to SF [153]. By reducing inflammatory disruption of sleep-regulating neural circuits and limiting nocturnal autonomic overactivation, T3 may help restore physiological conditions that favor CSF-ISF exchange and perivascular waste removal [154]. From a preventive standpoint, T3 may be especially relevant in individuals with early-stage CSVD, cardiometabolic comorbidities, or age-related SF, in whom glymphatic inefficiency and endothelial dysfunction precede irreversible structural brain injury [134]. Nutritional interventions targeting these upstream vulnerabilities could complement conventional vascular risk management strategies and potentially delay the onset or progression of cognitive impairment (Figure 6).

Despite this strong mechanistic rationale, definitive confirmation of T3 efficacy within the sleep–glymphatic–vascular axis will require targeted experimental and clinical studies. Priority outcomes should include assessments of AQP4 polarization and anchoring complex integrity (Dmd–Dag–Snta1), in vivo glymphatic function using DTI-ALPS, structural markers such as ePVS and WMHs, and objective sleep metrics derived from polysomnography or actigraphy. Collectively, tocotrienols represent a biologically plausible, yet still unproven, nutraceutical strategy for targeting upstream contributors to glymphatic dysfunction and CSVD progression. Their documented antioxidant, anti-inflammatory, endothelial-protective, and neurovascular-stabilizing properties support a strong mechanistic rationale for further investigation. However, direct causal evidence linking tocotrienol supplementation to improvements in glymphatic flux, perivascular clearance, or CSVD lesion burden remains absent, highlighting an important translational gap for future preclinical and clinical studies. Figure 6 integrates the preceding mechanistic (Figure 2) and pathophysiological (Figure 4) layers into a systems pharmacology model, highlighting potential therapeutic entry points through tocotrienol-mediated network modulation across sleep regulation, astrocytic function, and neurovascular integrity.

## 7. Current Evidence from Preclinical and Clinical Studies

### 7.1. Animal Models of Vascular Injury and Sleep Disruption

As discussed in previous sections, a growing body of preclinical evidence demonstrates that vascular risk factors and sleep disturbances converge on shared neurovascular and glymphatic pathways that are central to CSVD pathogenesis. Rodent models of aging consistently exhibit hallmark features of vascular dysfunction, including increased BBB permeability, downregulation of tight junction proteins (e.g., claudin-5, occludin, ZO-1), and reductions in CBF [155]. These alterations collectively impair perivascular homeostasis and promote white matter vulnerability characteristic of early CSVD. Hypertensive rodent models further reveal endothelial dysfunction marked by reduced NO bioavailability, enhanced oxidative stress, and heightened inflammatory signaling [156]. These changes compromise BBB integrity and promote perivascular inflammation, mirroring human hypertensive arteriopathy. Similarly, models of chronic cerebral hypoperfusion and diabetes demonstrate profound disruption of endothelial tight junctions and pericyte coverage, driven by ROS, pro-inflammatory cytokine release, and activation of advanced glycation end products (AGEs) binding to their receptor (RAGE) (AGE–RAGE) signaling pathways [157,158]. Genetic models, including NOTCH3 mutant mice and smooth muscle-specific Mypt1 knockout (MYPT1^SMKO^) mice, provide mechanistic insight into hereditary CSVD, illustrating how intrinsic vascular vulnerabilities interact with metabolic and environmental stressors to destabilize endothelial–pericyte coupling and precipitate BBB breakdown [159,160].

Parallel lines of investigation highlight sleep disruption as a potent amplifier of vascular and glymphatic pathology. Chronic SF in both transgenic and wild-type rodents increases amyloid-β and tau accumulation, activates microglia and astrocytes, and induces deficits in learning and memory [36,47]. In models of traumatic brain injury, SF exacerbates hippocampal microglial activation, intensifies pro-inflammatory cytokine production, and worsens neuronal dysfunction, underscoring the synergistic effects of sleep loss on neurovascular and neuroimmune pathways [61,161]. Mechanistic studies consistently link these outcomes to impaired glymphatic clearance, characterized by mislocalization of astrocytic AQP4 away from perivascular end feet, disruption of the dystrophin–glycoprotein anchoring complex due to dystrophin 17 deficiency [58], and dysfunction of endosome–autophagosome–lysosome pathways [36]. Restoration of sleep architecture or selective enhancement of SWS in rodent models improves glymphatic flux, reduces neurotoxic protein accumulation, and attenuates neuroinflammation, providing compelling evidence for a causal role of sleep in maintaining glymphatic and vascular health [162].

### 7.2. Human Evidence on Vascular Aging, Cognition, and Inflammation

Human studies increasingly provide indirect but converging evidence supporting the relevance of glymphatic dysfunction and sleep disturbances to vascular cognitive impairment and CSVD. Advanced neuroimaging techniques, particularly DTI-ALPS, alongside structural markers such as ePVS, have emerged as non-invasive proxies for glymphatic integrity. Reduced DTI-ALPS indices in individuals with Parkinson’s disease, post-stroke syndromes, and subjective cognitive complaints correlate with impaired cognition, white matter abnormalities, and markers of vascular dysfunction [31,163]. Sleep quality appears to be a critical modulator of these associations. Self-reported sleep disturbances, commonly assessed using the PSQI, are consistently associated with increased ePVS burden and poorer white matter integrity, suggesting that insufficient or SF negatively impacts perivascular clearance in humans [46,68]. Although these studies are largely observational, they reinforce the translational relevance of preclinical findings linking sleep, glymphatic dysfunction, and vascular pathology.

From an interventional perspective, T3 represent a biologically plausible and clinically accessible strategy to target the sleep–glymphatic–vascular axis. Although randomized controlled trials (RCTs) remain limited, emerging clinical data suggest potential cognitive and sleep-related benefits. In a 12-week supplementation study, daily administration of 100 mg T3 improved overall memory performance, particularly non-verbal memory, while also reducing self-reported sleep disturbances and modulating inflammatory markers [164]. Importantly, T3 was well tolerated, with no serious adverse events reported, supporting its feasibility for long-term preventive use. However, current human evidence is constrained by heterogeneity in cognitive and sleep assessments, reliance on indirect glymphatic biomarkers, and a paucity of longitudinal data. Most glymphatic studies to date involve individuals with established neurological disorders, limiting causal inference and obscuring early disease mechanisms [27,165].

Future clinical applications should prioritize integrative trial designs incorporating objective sleep measures (e.g., polysomnography or actigraphy), neuroimaging markers of glymphatic function (DTI-ALPS, ePVS), vascular integrity (BBB permeability, endothelial biomarkers), and inflammatory profiles. Stratification by vascular risk factors, metabolic comorbidities, and sleep quality may help identify subpopulations most likely to benefit from tocotrienol-based interventions. Such approaches could establish tocotrienols not merely as cognitive supplements, but as multimodal modulators of neurovascular resilience with relevance for CSVD prevention and progression. Table 1 summarizes the translational convergence between preclinical models of vascular injury and sleep disruption and corresponding human neuroimaging, molecular, and functional biomarkers relevant to cerebral small vessel disease. The table highlights shared mechanistic pathways linking glymphatic dysfunction, neurovascular injury, and inflammation, and maps these processes to putative tocotrienol targets within the sleep–glymphatic–vascular continuum.

### 7.3. T3 Studies Relevant to Neurovascular Health

Accumulating preclinical and emerging clinical evidence supports T3 as a multi-target neurovascular modulator with mechanistic relevance to CSVD and glymphatic dysfunction. Across diverse experimental platforms, T3 consistently attenuates oxidative stress, suppresses neuroinflammatory signaling, and preserves vascular and neuronal integrity, processes that converge on the pathophysiology of SF–induced glymphatic failure. Importantly, these effects extend beyond classical antioxidant activity and encompass astrocytic, microglial, and endothelial pathways central to perivascular clearance and small vessel health.

At the cellular level, T3 demonstrates robust anti-inflammatory effects in microglial models. Recent studies integrating network pharmacology with experimental validation in neurological disorders have demonstrated that pathway-level predictions gain translational credibility when coupled with in vivo or cellular confirmation of target engagement and phenotypic rescue [166,167,168]. Incorporating such hybrid approaches into future T3 research could move mechanistic claims beyond in silico plausibility toward experimentally grounded systems pharmacology. Studies using BV2 microglia show that δ-T3 suppresses NO production, IL-1β release, and 5-lipoxygenase signaling, thereby limiting the propagation of neuroinflammatory cascades that impair endothelial function and astrocytic polarity [169]. These findings are particularly relevant given the established role of chronic microglial activation in disrupting BBB integrity and promoting AQP4 depolarization, key contributors to glymphatic inefficiency in CSVD. In parallel, neuronal culture studies indicate that α-T3 mitigates oxidative stress–induced neurite degeneration and inhibits microtubule affinity-regulating kinase (MARK)-dependent tau phosphorylation, linking T3 activity to reduced cytoskeletal instability and downstream neurovascular stress [170].

Moreover, in vivo, TRF has shown consistent benefits across rodent models of vascular dementia, aging, and neurodegeneration. In aluminum chloride (AlCl_3_)–induced and age-associated vascular dementia models, TRF improves endothelial-dependent vasodilation, reduces myeloperoxidase activity, and enhances platelet-derived growth factor C (PDGF-C)–mediated vascular repair, culminating in improved cognitive outcomes [136]. These vascular effects are highly relevant to glymphatic function, as endothelial dysfunction and impaired arterial pulsatility are known to reduce perivascular CSF influx and waste clearance. Similarly, in transgenic Alzheimer’s disease models, T3 reduces amyloid burden and preserves neuronal architecture [171], which may indirectly alleviate glymphatic load and perivascular congestion that exacerbates small vessel pathology.

Beyond vascular dementia and Alzheimer’s disease paradigms, T3 also demonstrates neuroprotective efficacy in toxin-induced Parkinsonian models, where δ-T3 preserves dopaminergic neurons and suppresses inflammatory signaling [144]. Although glymphatic outcomes were not directly assessed in these studies, the attenuation of neuroinflammation and oxidative stress targets upstream drivers of perivascular dysfunction common to multiple neurodegenerative conditions. Human evidence, while still limited, provides preliminary translational support. A randomized supplementation study in adults with subjective memory complaints reported improvements in overall and non-verbal memory, reductions in inflammatory markers (e.g., TNF-α), and decreased self-reported sleep disturbances following 12 weeks of tocotrienol intake [118,172]. These findings are notable given the established links between sleep quality, systemic inflammation, glymphatic efficiency, and CSVD risk. Importantly, T3 was well tolerated, supporting its feasibility as a long-term nutritional intervention.

Despite these promising findings, several limitations remain. Oral bioavailability of T3 is variable and influenced by formulation, lipid co-administration, and BBB integrity [110,173]. Moreover, direct evidence linking T3 supplementation to improvements in AQP4 polarization, perivascular clearance, ePVS burden, or DTI-ALPS metrics is currently lacking. Addressing these gaps will require integrative experimental designs combining sleep phenotyping, advanced neuroimaging, and molecular analyses of astrocytic–vascular interfaces. Overall, the studies summarized in Table 2 highlight T3 as a biologically plausible, multi-level modulator of neurovascular health. By simultaneously targeting oxidative stress, inflammation, endothelial dysfunction, and sleep-related upstream processes, tocotrienols occupy a unique position within the sleep–glymphatic–vascular framework. These properties warrant further investigation in mechanistic preclinical models and well-powered clinical trials incorporating glymphatic-specific endpoints to clarify their role in preventing or slowing CSVD progression.

### 7.4. Limitations of Existing Studies

Despite compelling mechanistic and emerging clinical evidence, several limitations constrain the interpretation and translational relevance of current findings linking SF, glymphatic dysfunction, CSVD, and T3 intervention. A substantial proportion of mechanistic insights derives from rodent models, which differ from humans in cerebrovascular architecture, white matter composition, sleep structure, and glymphatic dynamics. These interspecies differences, particularly in perivascular space geometry, astrocytic AQP4 distribution, and arterial pulsatility [174], limit direct extrapolation to human CSVD pathophysiology and underscore the need for improved translational models. Current human evidence linking sleep disturbance to glymphatic dysfunction and CSVD relies predominantly on observational associations using indirect imaging and behavioral proxies, including DTI-ALPS indices, enlarged perivascular spaces, and subjective or actigraphy-based sleep measures [175]. These approaches, while informative, are inherently limited by residual confounding from age, vascular comorbidities (e.g., hypertension, diabetes, dyslipidaemia), medication use, and co-existing neurodegenerative pathology, all of which independently influence perivascular clearance efficiency and CSVD burden. Consequently, reported associations cannot be interpreted as causal and should be viewed as hypothesis-generating. Additionally, most available human studies are cross-sectional, precluding robust inference regarding the temporal directionality between sleep fragmentation, glymphatic impairment, and CSVD progression. It therefore remains unclear whether sleep disruption primarily drives perivascular clearance failure, whether early microvascular pathology impairs sleep–glymphatic coupling, or whether both processes reflect shared upstream neurovascular vulnerability. Longitudinal and interventional designs are required to disentangle these bidirectional relationships.

Translational interpretation is further constrained by substantial methodological heterogeneity across studies, including variability in sleep assessment tools (subjective questionnaires, actigraphy, polysomnography), imaging acquisition and analysis pipelines for DTI-ALPS, and rating scales for ePVS burden. Techniques such as DTI-ALPS and MRI-based quantification of ePVS offer valuable in vivo proxies but remain sensitive to vascular pulsatility, ISF shifts, and regional white matter integrity [75]. Direct visualization of CSF-ISF exchange in humans remains technically challenging, emphasizing the need for multimodal imaging strategies that integrate diffusion metrics, perfusion measures, and sleep-state characterization [83]. Differences in scanner field strength, diffusion protocols, region-of-interest placement, and inter-rater reliability further limit cross-study comparability and may contribute to effect size variability. In the context of T3 research, additional limitations include variability in formulations, dosing regimens, and outcome measures across studies. Poor oral bioavailability and rapid hepatic metabolism of T3 further complicate clinical translation, potentially limiting brain exposure despite robust preclinical efficacy [173]. Importantly, no studies to date have directly evaluated the effects of tocotrienols on glymphatic markers such as AQP4 polarization, perivascular space dynamics, or DTI-ALPS indices in either animal or human models.

Collectively, these observational findings provide a biologically plausible rationale for exploring sleep-targeted interventions as a potential strategy to support perivascular clearance in CSVD; however, direct evidence of clinical efficacy remains lacking and requires validation in prospective, standardized, and interventional human studies, these gaps highlight several priorities for future research: (i) longitudinal human studies integrating objective sleep metrics with glymphatic imaging and vascular biomarkers; (ii) experimental designs that directly assess astrocytic polarity, dystrophin-associated anchoring complexes, and perivascular clearance pathways; (iii) development of optimized T3 formulations with enhanced bioavailability and brain penetration, and (iv) future translational progress will require harmonized protocols combining polysomnography, advanced diffusion-based glymphatic metrics, dynamic contrast-enhanced imaging of perivascular flow, and longitudinal tracking of CSVD markers, alongside careful control for vascular comorbidities and medication effects. Addressing these limitations will be essential for translating the emerging sleep–glymphatic–vascular framework into effective preventive and therapeutic strategies for cerebral small vessel disease.

### 7.5. Current Gaps, Future Directions, and Clinical/Public Health Implications

Despite rapid advances in understanding the sleep–glymphatic–vascular continuum, several critical knowledge gaps remain that limit translation into clinical practice. First, causal relationships between SF and glymphatic dysfunction in humans are still largely inferential. Most available data rely on cross-sectional sleep assessments and indirect imaging proxies, such as DTI-ALPS or perivascular space burden, without concurrent evaluation of sleep architecture, cerebrovascular pulsatility, and astrocytic polarity [83]. Future studies must integrate longitudinal human studies combining objective sleep metrics with polysomnography, vascular hemodynamic measures, and glymphatic imaging (i.e., DTI-ALPS/ePVS) to disentangle directionality and temporal sequencing. Interventional sleep trials (CBT-I, OSA treatment) with glymphatic imaging endpoints are also suggested.

Second, the molecular regulation of AQP4 polarization in human disease remains insufficiently characterized. While preclinical studies implicate dystrophin–dystroglycan–syntrophin (Dmd–Dag–Snta1) complex and inflammatory signaling in AQP4 mislocalization, validation in aging humans and CSVD cohorts is lacking. High-resolution post-mortem studies and in vivo biomarkers reflecting astrocytic polarity and perivascular integrity are urgently needed. Third, the interaction between vascular stiffness, arterial pulsatility, and sleep-dependent glymphatic transport remains poorly defined. Aging, hypertension, and metabolic disorders alter cerebrovascular compliance, yet how these changes modify CSF–ISF exchange across sleep–wake cycles is unclear. Multimodal studies incorporating arterial stiffness indices, nocturnal blood pressure dipping, and sleep-stage–specific glymphatic measures represent an important future direction.

Finally, from a nutritional perspective, direct evidence linking T3 to glymphatic modulation is currently absent. While T3 exhibits robust antioxidants, anti-inflammatory, and endothelial-protective properties, its effects on AQP4 polarization, perivascular space dynamics, and sleep architecture remain speculative [176]. Future preclinical studies should directly assess these endpoints, while early-phase clinical trials should incorporate objective sleep metrics and glymphatic imaging. Additionally, advances in formulation science, including nano-emulsions, lipid carriers, or combination therapies, will be critical to overcome bioavailability limitations and maximize neurovascular delivery. Formulation-optimized T3 trials with CNS exposure biomarkers are also suggested.

Moreover, the convergence of SF, glymphatic dysfunction, and CSVD has substantial clinical and public health implications, particularly in aging populations. CSVD is a leading contributor to vascular cognitive impairment and dementia, yet remains largely underdiagnosed until irreversible white matter damage occurs [177]. The emerging recognition of sleep disruption as a modifiable upstream risk factor provides a valuable opportunity for early intervention. From a clinical standpoint, routine assessment of sleep quality and fragmentation should be considered in individuals at risk for CSVD, including older adults, patients with hypertension, diabetes, and OSA, and those with subjective cognitive complaints. Integration of sleep metrics with neuroimaging markers such as ePVS and DTI-ALPS may enhance risk stratification and enable earlier identification of glymphatic and vascular vulnerability. In addition, mechanistic animal studies testing whether T3 alters perivascular clearance dynamics is of important.

Nutritional strategies represent a particularly attractive adjunct in this context. T3, as bioactive vitamin E isoforms, offers a low-cost, generally safe, and potentially scalable intervention that targets oxidative stress, neuroinflammation, endothelial dysfunction, and possibly sleep regulation, key drivers of CSVD progression. Systems pharmacology pipelines with experimental validation are suggested. While not a substitute for established vascular risk management, T3 supplementation could complement lifestyle interventions such as sleep optimization, physical activity, and metabolic control. At the population level, these findings underscore the importance of sleep health as a public health priority, comparable to diet and cardiovascular risk factor management. Public awareness initiatives emphasizing sleep continuity, alongside nutritional approaches that support neurovascular resilience, may contribute to reducing the long-term burden of vascular cognitive impairment and dementia. Future studies integrating polysomnography, DTI-ALPS, perivascular space imaging, and molecular assessment of astrocytic AQP4 polarization in T3-treated models are essential to move this field from mechanistic plausibility to empirical validation. Ultimately, integrating sleep, nutrition, and vascular health within a unified preventive framework could shift CSVD management from late-stage intervention toward early, mechanism-informed prevention. Finally, while this review integrates converging evidence across sleep biology, glymphatic neuroscience, and nutritional neurovascular protection, we emphasize that the proposed tocotrienol–glymphatic links remain hypothesis-driven and should be interpreted as a conceptual framework to guide experimental design rather than as definitive mechanistic conclusions.

## 8. Conclusions

CSVD emerges from the convergence of vascular dysfunction, impaired brain waste clearance, and disrupted sleep architecture. Accumulating evidence supports a unifying framework in which SF acts as a critical upstream stressor that destabilizes glymphatic transport, promotes astrocytic and endothelial dysfunction, and accelerates small vessel pathology. Within this sleep–glymphatic–vascular continuum, impaired SWS, AQP4 depolarization, perivascular space enlargement, and neuroinflammation form interconnected mechanisms that contribute to white matter injury, cognitive decline, and increased dementia risk. T3, as bioactive vitamin E isoforms, occupy a distinctive position within this framework. Their capacity to modulate oxidative stress, neuroinflammatory signaling, endothelial function, and mitochondrial homeostasis provides a compelling mechanistic rationale for targeting multiple upstream nodes of CSVD pathogenesis. However, direct evidence linking T3 to glymphatic modulation or disease modification in humans remains limited, and current support for their role in this context is primarily inferential and hypothesis-driven. Existing preclinical and emerging clinical observations nonetheless suggest that T3 may influence early, potentially reversible neurovascular and neuroglial perturbations associated with age-related SF and cardiometabolic vulnerability. Importantly, this integrative perspective reframes CSVD not solely as a vascular disorder, but as a systems-level condition shaped by sleep physiology, neuroimmune signaling, and nutritional status. Future research priorities include longitudinal human studies combining objective sleep assessment, harmonized glymphatic imaging, and rigorous control of vascular confounders, alongside mechanistic validation in experimental models and pharmacokinetic optimization of tocotrienol formulations. Collectively, such efforts are required to move from mechanistic plausibility toward evidence-based translational application, and to determine whether T3 can be positioned as a safe, scalable adjunct within broader preventive strategies for preserving neurovascular health and mitigating CSVD-related cognitive decline.

## Figures and Tables

**Table 1:** Translational mapping of preclinical models to human biomarkers and tocotrienol (T3) targets.

Domain	Animal Models/Evidence	Key Mechanisms Identified	Human Biomarkers/Readouts	Putative T3 Targets (T3/TRF) and LIMITATIONS
Aging-related CSVD [155]	In vivo animal (Aged rodents); observational human	BBB leakage ↓ tight junctions ↓ CBF AQP4 depolarization	WMHs and ePVS burden ePVS DTI-ALPS index ↓	Antioxidant (Nrf2) Endothelial NO preservation Astrocytic redox stability Limitation: no direct intervention linking T3 to glymphatic restoration; ALPS/ePVS are indirect proxies
Hypertension models [156]	In vivo animal (SHR, Ang-II infusion); observational human	Endothelial dysfunction Oxidative stress Perivascular inflammation	Pulse wave velocity WMHs BBB permeability markers	NF-κB inhibition NO bioavailability Vascular stiffness reduction Limitations: vascular outcomes are associated in humans; confounding by age/comorbidities
Chronic cerebral hypoperfusion [157]	In vivo animal (bilateral carotid stenosis); observational human	Pericyte loss TJ disruption Glymphatic failure	DTI-ALPS index ↓ White matter FA ↓	Endothelial protection Anti-inflammatory signaling Limitations: no direct human evidence for glymphatic rescue; ALPS reflects diffusion properties, not flux
Diabetes/metabolic models [158]	In vivo animal (STZ-induced diabetes); observational human	AGE–RAGE activation Oxidative BBB injury	Insulin resistance CSVD MRI burden	Mitochondrial protection Lipid peroxidation reduction Limitations: metabolic confounding in humans; unclear CNS bioavailability of T3
Genetic CSVD models [159,160]	In vivo genetic models (NOTCH3 mutants and MYPT1^SMKO^); clinical imaging correlation	Endothelial–pericyte uncoupling BBB breakdown	Lacunes; microbleeds; WMHs	Endothelial signaling modulation Limitation: monogenic models may not reflect sporadic CSVD biology
Sleep fragmentation [36,47]	In vivo animal (mechanical sleep disruption); cross-sectional human sleep studies	AQP4 mislocalization ↓ Glymphatic flux ↑ Aβ/tau	PSQI score ↑ ePVS ↑ DTI-ALPS index ↓	Sleep stabilization via neuroinflammation attenuation Limitations: human sleep measures are subjective; OSA confounded by hypoxia and BP surges
Neuroinflammation [61,161]	In vivo animal (LPS); observational human biomarkers (TBI + sleep loss)	Microglial pro-inflammatory shift (Iba1, CD68, iNOS)	Plasma IL-6 and TNF-α TSPO-PET	NF-κB suppression Microglial repolarization Limitations: TSPO-PET lacks cell specificity; peripheral cytokines are non-specific
Astrocytic polarity [36]	In vivo animal (AQP4 knockout or depolarization models); indirect human imaging	Loss of Dmd–Dag–Snta1 anchoring	Indirect via DTI-ALPS ePVS	Preservation of dystrophin–glycoprotein complex Limitations: no direct in vivo human assessment of AQP4 polarity

↓ indicate decrease/reduced/lower; ↑ indicate increased/higher. AQP4, aquaporin-4; BBB, blood–brain barrier; CBF, cerebral blood flow; CSF, cerebrospinal fluid; CSVD, cerebral small vessel disease; DTI-ALPS, diffusion tensor imaging–analysis along the perivascular space; ePVS, enlarged perivascular spaces; ISF, interstitial fluid; NF-κB, nuclear factor kappa-B; ROS, reactive oxygen species; SWS, slow-wave sleep; TJ, tight junction; TRF, tocotrienol-rich fraction; WMHs, white matter hyperintensities.

**Table 2:** Preclinical and clinical evidence supporting tocotrienols (T3) as modulators of neurovascular, inflammatory, and glymphatic-relevant pathways.

Model	T3 Intervention	Targeted Pathways	Outcomes	Relevance to Glymphatic-CSVD	Ref.
BV2 microglia cell culture	δ-T3 (palm oil-derived)	Attenuation of NO, IL-1β, and 5-LOX signaling	Reduced microglial inflammatory mediator production	Neuroinflammation contributes to endothelial dysfunction and BBB compromise in CSVD Reducing inflammatory burden may indirectly support vascular-glymphatic function	[20,169]
Neuronal cells under oxidative stress	α-T3 (5–10 μM)	Inhibition of MARK activation. Reduction in oxidative stress.	Prevention of neurite degeneration Decreased pathological tau phosphorylation	Oxidative and tau-related neuronal injury exacerbates neurovascular stress Neuronal protection may reduce subsequent vascular and glymphatic impairment	[170]
AlCl_3_-induced vascular dementia rat model	T3-rich fraction (TRF)	Antioxidant activity. Reduced MPO Activation of PDGF-C signaling	Improved endothelial function Reduced vascular inflammation Improved cognition	Endothelial dysfunction is a key feature of CSVD Vascular protection may preserve perivascular pathways relevant to glymphatic function	[135]
Ageing and vascular dementia rodent models	TRF (various formulations)	Oxidative stress reduction Promotion of PDGF-C-mediated repair	Improved cognitive performance and neurovascular markers across models	Synthesize preclinical evidence supporting T3 as a neurovascular protectant relevant to CSVD mechanisms	[136]
Transgenic AD rodent models	TRF and α-T3	Antioxidant activity Reduction in amyloid deposition	Moderate cognitive improvement Reduced amyloid burden	Protein accumulation exacerbates glymphatic stress T3 effects may reduce pathological load relevant to CSVD	[128,171]
MPTP-induced PD mouse model	δ-T3	Anti-inflammatory and neuroprotective mechanisms	Preservation of dopaminergic neurons Improved motor function	Neuroinflammation and vascular dysfunction impair glymphatic efficiency T3 may attenuate upstream contributors	[20]
Adults with subjective memory complaints (40–80 y)	T3 (100 mg/day, 12 weeks)	Modulation of TNF-α and reduction in oxidative stress (MDA)	Improved overall and non-verbal memory Reduced self-reported sleep disturbance, and good tolerability	Sleep quality and inflammation influence glymphatic efficiency and CSVD risk Provides preliminary human evidence linking tocotrienols to upstream modulators	[164]

β, amyloid-β; BBB, blood–brain barrier; CSVD, cerebral small vessel disease; IL, interleukin; MARK, microtubule affinity-regulating kinase; MPO, myeloperoxidase; NO, nitric oxide; PDGF-C, platelet-derived growth factor-C; ROS, reactive oxygen species; TRF, tocotrienol-rich fraction.

## Data Availability

Not applicable. No new data were created or analyzed in this study.

## Abbreviations

The following abbreviations are used in this manuscript:

AlCl_3_	Aluminum chloride
Ang-II	Angiotensin II
AQP4	Aquaporin-4
Arg1	Arginase-1
BBB	Blood–brain barrier
CAT	Catalase
CBF	Cerebral blood flow
CBV	Cerebral blood volume
CMBs	Cerebral microbleeds
COX-2	Cyclooxygenase-2
CSF	Cerebrospinal fluid
CSVD	Cerebral small vessel disease
DAMPs	Damage-associated molecular patterns
Dmd–Dag–Snta1	Dystrophin–glycoprotein–syntrophin anchoring complex
DTI-ALPS	Diffusion tensor imaging–analysis along the perivascular space
ePVS	Enlarged perivascular spaces
GPx	Glutathione peroxidase
HPA	Hypothalamic–pituitary–adrenal
IL	Interleukin
iNOS	Inducible nitric oxide synthase
ISF	Interstitial fluid
MARK	Microtubule affinity-regulating kinase
MPO	Myeloperoxidase
MYPT1^SMKO^	Smooth muscle-specific Mypt1 knockout
NF-κB	Nuclear factor kappa-light-chain-enhancer of activated B
NGVU	Neuro-glia-vascular unit
NIRS	Near-infrared spectroscopy
NO	Nitric oxide
NREM	Non–rapid eye movement
Nrf2	Nuclear factor erythroid 2–related factor 2
OSA	Obstructive sleep apnea
PDGF-C	Platelet-derived growth factor C
PKC	Protein kinase C
PSQI	Pittsburgh Sleep Quality Index
RCTs	Randomized controlled trials
ROS	Reactive oxygen species
SF	Sleep fragmentation
SHR	Spontaneously hypertensive rat
SOD	Superoxide dismutase
SWS	Slow-wave sleep
T3	Tocotrienols
TBI	Traumatic brain injury
TJ	Tight junction
TNF-α	Tumor necrosis factor-α
TRF	Tocotrienol-rich fractions
VEGF	Vascular endothelial growth factor
WMHs	White matter hyperintensities
α-T3, δ-T3	Alpha-tocotrienol, Delta-tocotrienol
